# Collaborative Indoor Positioning Systems: A Systematic Review

**DOI:** 10.3390/s21031002

**Published:** 2021-02-02

**Authors:** Pavel Pascacio, Sven Casteleyn, Joaquín Torres-Sospedra, Elena Simona Lohan, Jari Nurmi

**Affiliations:** 1 Institute of New Imaging Technologies, Universitat Jaume I, 12006 Castellón, Spain; sven.casteleyn@uji.es (S.C.); jtorres@uji.es (J.T.-S.); 2 Electrical Engineering Unit, Tampere University, 33014 Tampere, Finland; elena-simona.lohan@tuni.fi (E.S.L.); jari.nurmi@tuni.fi (J.N.); 3 UBIK Geospatial Solutions S.L., 12006 Castellón, Spain

**Keywords:** collaborative indoor positioning systems, positioning technique, positioning method, positioning technology, location-based services, systematic literature review

## Abstract

Research and development in Collaborative Indoor Positioning Systems (CIPSs) is growing steadily due to their potential to improve on the performance of their non-collaborative counterparts. In contrast to the outdoors scenario, where Global Navigation Satellite System is widely adopted, in (collaborative) indoor positioning systems a large variety of technologies, techniques, and methods is being used. Moreover, the diversity of evaluation procedures and scenarios hinders a direct comparison. This paper presents a systematic review that gives a general view of the current CIPSs. A total of 84 works, published between 2006 and 2020, have been identified. These articles were analyzed and classified according to the described system’s architecture, infrastructure, technologies, techniques, methods, and evaluation. The results indicate a growing interest in collaborative positioning, and the trend tend to be towards the use of distributed architectures and infrastructure-less systems. Moreover, the most used technologies to determine the collaborative positioning between users are wireless communication technologies (Wi-Fi, Ultra-WideBand, and Bluetooth). The predominant collaborative positioning techniques are Received Signal Strength Indication, Fingerprinting, and Time of Arrival/Flight, and the collaborative methods are particle filters, Belief Propagation, Extended Kalman Filter, and Least Squares. Simulations are used as the main evaluation procedure. On the basis of the analysis and results, several promising future research avenues and gaps in research were identified.

## 1. Introduction

The advent of mobile computing, including Internet of Things (IoT) and wearable devices, has changed the traditional scope of positioning systems, which moved from military tracking and civilian navigation to location information [1]. Location information is a key element to bridge the gap between the physical and the digital world, either for personal [2,3,4,5] or industrial use [6,7,8,9].

The new generation of smart applications with location-based service (LBSs) are part of our daily lives. They help us to get the best route to our work place using crowdsourced information [10], find a restaurant based on our preferences [11] or, even, remember preventive measures in risky situations for our health [12]. Undoubtedly, precise positioning plays a key role in LBS.

In outdoor environments, Global Navigation Satellite System (GNSSs)—e.g., Global Positioning System (GPS), Globalnaya Navigazionnaya Sputnikovaya Sistema (GLONASS), Galileo, and BeiDou [13]—are widely adopted for global positioning purposes [14,15]; i.e., GNSS is supposed to provide accurate positioning anywhere on Earth. The GPS accuracy, in terms of positioning error, in smartphones is usually within a 4.9 m radius in clear open sky conditions, but it is capable of centimeter accuracy when it is used in combination with dual-frequency receivers and/or augmentation systems [16,17]. Despite the high accuracy and global coverage provided by GNSSs, they cannot properly operate indoors. The strong signal attenuation, the presence of heavy signal multipath, and other sources of interference invalidate GNSS as a positioning solution indoors [14,18].

In contrast to outdoor environments, indoor environments present diverse and dynamic scenarios with complex geometries. The indoor environments are heterogeneous and include homes, offices, warehouses, hospitals, and shopping malls, among many others. Furthermore, the applications for end-users are diverse and, therefore, they have different accuracy and coverage requirements. As pointed out by Mautz [19], Ambient Assisted Living (AAL) applications require room-level coverage with accuracy below 1 m, whereas law-enforcement applications have urban/rural coverage with accuracy of a few meters. Therefore, the particular characteristics of the indoor scenarios and the diversity of the applications has made that no single Indoor Position System (IPS) has emerged as a universal solution.

The available indoor solutions are highly coupled to the environment and target application. We can find, for instance, smart home systems designed to help us locate misplaced objects using 802.15.4a [20]; systems to monitor the daily activities of seniors at home using smartwatches and IEEE 802.11 Wireless LAN (Wi-Fi) fingerprinting [21]; or remote patient monitoring with ZigBee [22]. Not only the coverage and accuracy are important to select the base positioning technology, the deployment and maintenance costs are also relevant. The diverse solutions are a clear indicator that there is no single alternative for GNSS indoors, and different positioning technologies co-exist.

In addition to specific requirements of the scenarios and applications, the IPSs must also cover different requirements depending on the kind of actors of the system, which include aerial robots [23,24], mobile terrestrial robots [25,26,27], and humans [28,29]. These actors present diverse needs, as robots require accurate positioning to achieve safe autonomous operation, whereas the IPSs focused on human tasks are not required to perform control actions. Furthermore, in contrast to IPSs for robots, those for humans are usually restricted to devices already in use by the user (e.g., smartphone, smart watch), which inherently imposes battery and computational power constraints. In this article we focus on Collaborative Indoor Positioning System (CIPS) for humans.

Advanced solutions based on the combination of multiple positioning technologies have also been widely used [30,31,32,33]. For instance, ref. [34] introduce an application that combines Bluetooth Low Energy (BLE) and Pedestrian Dead Reckoning (PDR) with a particle filter with the purpose of guiding people with visual impairments. As stated in [32], sensor fusion efficiently combines data from disparate sources (e.g., sensors) to generate better information than that reported by the original sources individually. Combining multiple sensors for positioning can minimize their constraints: low frequency in Wi-Fi, unpredictable external disturbances affecting the magnetometer [35], fluctuation on the barometer [36], drifts in gyroscope [37], or random noise and bias present in Microelectro-Mechanical System (MEMS) and Inertial Measurement Units (IMUs) [38].

Within IPSs, collaborative positioning has become relevant in the last years. CIPSs might be considered the evolution of sensor fusion, as they also combine data from multiple sources. As a differentiating factor, CIPSs rely on various independent actors who share sensing information, conveying key positioning data from heterogeneous sensors, to enable the positioning of every actor and improve it along different dimensions [39].

CIPSs present some advantages over the conventional IPSs approaches. They expand the coverage area of stand-alone IPSs by sharing the position of users [40,41,42]. They reduce the use of expensive and/or complex positioning infrastructure while enhancing the position accuracy of users [43,44,45]. They reduce positioning ambiguities due to poor geometric location of anchors [46,47]. They also reduce positioning error in harsh and Non-line-of-sight (NLOS) environments using the surrounding users as auxiliary anchor nodes [48,49,50]. CIPSs have applied different technologies, techniques, and methods to address positioning and achieve the aforementioned advantages, yet a comprehensive overview of this emerging, diverse field is missing.

This paper introduces a systematic review on CIPSs. The review is based on the Preferred Reporting Items for Systematic Reviews and Meta-Analyses (PRISMA) guidelines [51]. We identify, analyze, classify, and discuss the main findings on CIPSs reported in the scientific literature indexed in Scopus or Web of Science datasets. Despite the publication of several surveys and reviews related to IPSs [52,53,54,55,56,57], none of them focuses on collaborative approaches. This article therefore focuses on the following:

1. Systematically collecting and analyzing research works related to CIPSs;
2. Identifying and classifying the technologies, techniques, and methods applied;
3. Identifying and classifying the computation architectures and infrastructures required for positioning;
4. Identifying and describing the types of evaluation performed;
5. Analyzing and discussing the results, in order to provide an overview of CIPS, and to uncover trends, challenges, and gaps in this research field.

The remainder of this work is structured as follows. Section 2 presents the background in terms of IPS and related technologies, techniques, and methods. Section 3 describes the research methodology applied to conduct this systematic review. Section 4 graphically reports and analyzes the results. Section 5 further discusses the implications of the results in different dimensions. Additionally, the main limitations, current trends, and gaps are discussed. Finally, Section 6 summarizes the main findings and points out future work.

## 2. Background

In this section, we discuss relevant terminology, as well as existing classifications schemes for IPSs and their applied technologies, techniques, and methods. In addition, we present an overview of the CIPS, highlighting its advantages with respect to traditional IPS.

### 2.1. Indoor Positioning Systems

As its name indicates, an IPS is used to provide a position estimate in indoor environments. However, the design of an IPS highly depends on the context, and it is built on top of three main components. First, the base indoor positioning technology is the core of the IPS and will somehow be an indicator of the deployment’s context, i.e., the expected accuracy as well as any additional requirements and restrictions. In contrast to outdoor positioning, where synchronized, timestamped radio signals are transmitted from a constellation of satellites along a line of sight to the receiver, the indoor positioning technologies are of diverse nature and include well-known optical (e.g., Visible Light Communication (VLC)), radio frequency (e.g., Frequency Modulation (FM), Wi-Fi, BLE, among others), acoustic, and inertial measurement technologies. Second, the indoor positioning technique indicates what data/measurements or information are processed to calculate the position. For instance, the direction and angle from which a signal is received (Angle of Arrival (AoA)), the elapsed time of a signal from a transmitter to the receiver (Time of Arrival (ToA) and variants), the properties of the channel in a communication link (Channel State Information (CSI)), the strength of the signal at the receiver side (Received Signal Strength Indicator (RSSI)), or even the set of RSSIs from multiple emitters as a block (fingerprint) are mentioned. Third, and finally, the indoor positioning method is the particular algorithm used to process the data/measurements or information collected for positioning. In literature, a wide range of methods is described, from very particular variants of well-known algorithms (e.g., *k*-Nearest Neighbors (*k*-NN)), to only vaguely outlined methods which are referred to by the technique they use (e.g., fingerprint-based method). In addition, the indoor positioning methods can be specific for a particular technology and technique (e.g., PDR for inertial measurements), or they can be universal algorithms (e.g., Machine Learning algorithms such as *k*-NN or Support Vector Machines). To sum up, even though an IPS can be relatively simple, such as applying the *k*-NN algorithm over fingerprints of Wi-Fi signals [21,58,59], most of the advanced systems are complex, e.g., applying Extended Kalman Filters (EKFs) or particle filters to combine PDR over IMU data and fingerprinting based on ble [34,60].

Based on the computational architecture, the IPS can be classified into two categories: server-based—as in Where@UM [61]—and server-less/stand-alone—as in *AnyPlace* [62]—which indicate where the position estimate is computed. In a server-based architecture, the server processes the raw data provided by each device, without using information of the other devices, i.e., all the localization estimations are carried out in a remote server. In the server-less architecture, each device acquires the raw relevant data from sensors and processes them to self-determine the position, i.e., all the localization estimations are carried out locally on the device. In both cases, the position of a device is estimated using the data and information provided by that device.

Regarding the infrastructure, literature generally distinguishes between infrastructure-less and infrastructure-based IPS [63,64,65,66]. The infrastructure-less systems do not require to deploy any infrastructure in the area to operate, e.g., IPSs based on magnetic field [66]. In contrast, infrastructure-based IPSs require an infrastructure to operate, i.e., one or more physical elements deployed in the environment [63,65,66] (e.g., ble beacons or ultrasound receivers). To differentiate the systems where infrastructure needs to be purposely deployed versus systems that use existing infrastructure (i.e., signals of opportunity), some authors identified an in-between class: opportunistic IPS [67,68,69]. For example, IPSs based on Wi-Fi are considered opportunistic, if the environment is not altered to allow their operation (i.e., no Wi-Fi Access Points (APs) are purposely deployed for the IPS). In this paper, we do not consider opportunistic approaches as a separate class. For detailed information on (non-collaborative) IPSs, we refer to the excellent recent reviews and surveys available in literature, such as [53,70,71].

### 2.2. Indoor Positioning Technologies

From a technological point of view, researchers have proposed a wide variety of solutions for indoor positioning in search of improved performance in various application scenarios. In the literature, technologies for indoor positioning have been widely described, classified, used, and evaluated [14,19,52,53,54,55]. Nevertheless, a unified classification of the technologies is still missing. For instance, ref. [55] categorize technologies into six groups based on the kind of signal used to measure the position, hereby only covering Wireless Personal Networks. Authors in [19] classify the technologies into thirteen sensor technologies, based on the underlying idea that the performance of systems with the same type of sensors can be easily compared; similarly [53], in their meta-review, identify and describe ten categories to cover the most common technologies used in IPSs based on the type of sensors used. Authors in [14] summarize the specifications and features of twenty positioning technologies encountered in their survey, and they provide a categorization of the most suitable positioning technology already available for LBS applications. Table 1 summarizes the aforementioned classifications.

### 2.3. Indoor Positioning Techniques

The techniques applied to the IPS depend primarily on the technology used. Furthermore, the performance of the IPSs can vary dramatically depending on the type of technique applied, even when the technology and test conditions are identical. Therefore, in the literature, we find a significant number of works classifying and summarizing IPSs techniques and their features [14,52,53,54,55]. Similar as for technologies, techniques have been categorized from different points of view. Authors in Liu et al. [52] categorize techniques into three groups: the first group (Triangulation) is based on geometric properties to estimate the target position, which is divided in two subgroups (Lateration and Angulation), the Scene Analysis based on fingerprints measurements, and Proximity based on relative location information; Gu et al. [55] classify the techniques into four categories, and they add a new category (Vision Analysis) based on the image received by one or multiple points; Zafari et al. [54] in their classification do not create subgroups, and they present six techniques based on range measurements, one based on fingerprint, and a new one (Channel State information) based on the channel properties; Mendoza-Silva et al. [53] present a classification of four techniques based on the main three range measurements and AoA. Table 2 briefly presents a summary of some of the reported techniques.

### 2.4. Indoor Positioning Methods

The methods (also termed algorithms) for indoor positioning are defined as detailed sequences to follow in order to compute the position of a target object [55] and are intrinsically linked to the type of technologies and techniques used. Several research works that summarize them have been published, either generally, or specifically for a certain technology and/or technique. For example, for the latter, He and Chan [56] summarizes and classifies the methods used in Wi-Fi Fingerprinting based IPS as probabilistic or deterministic; Chen et al. [66] classifies the localization methods based on received Wi-Fi signal strength into geometric-based and fingerprinting-based schemes; Güvenc and Chong [57] provides an overview of ToA-based localization methods and classifies them into methods for Line-of-sight (LOS) and Non-line-of-sight (NLOS) scenarios; in contrast, Yassin et al. [72] conducts a general overview of methods covering the basic non-collaborative positioning techniques (Triangulation, Scene Analysis, and Proximity).

It is relevant to highlight that all these classifications are based on the operational phase of the IPS, not on the data collection phase (i.e., the origin of the reference data is not a factor). For instance, RADAR [58], the first fingerprint method, can still be applied to novel systems, whether the reference data are collected by means of crowdsourcing [73], obtained after interpolating a reduced radio map [74], automatically generated from unlabeled samples [75], or artificially generated by means of an advanced path-loss model [76]. Table 3 briefly presents a summary of some of the reported methods.

### 2.5. Collaborative Indoor Positioning Systems

Considering the role of different actors in IPSs, the literature distinguishes two main types: non-collaborative and collaborative [48,50,77,78]. This terminology refers to the operational phase (i.e., estimating the position), not the (reference) data gathering phase (e.g., building a fingerprint radio map). As such, non-collaborative schemes refer to systems that do not consider the participation of other users in their positioning algorithm [78]. In contrast, a CIPS is a scheme in which the position is determined based on the direct or indirect interaction between neighboring devices or diverse IPS. Note that collaborative approaches should not be confused with data or sensor fusion approaches. Whereas collaborative positioning is focused in systems whose independent actors (users or devices) exchange information and compute relative distances between them to provide the position of the set of users [77,78,79,80,81], sensor fusion combines information from various sensors from a single actor for providing the position of a single user [30,31,32,33].

Technological advances and the development of techniques and methods developed for traditional IPS are largely reused by collaborative systems to determine the position of collaborative nodes in CIPS. However, CIPS take advantage of those technologies that not only allow to estimate the position but also to exchange information between nodes. Within those technologies, we can distinguish wireless technologies (e.g., Wi-Fi, BLE, Ultra-wide band (UWB)) and cellular networks, which can be used with different well-known communication protocols, such as iBeacon and Bluetooth, among others. The methods of CIPSs are very diverse; however, some of the most studied methods are based on belief propagation and non-Bayesian approaches such as Least Square (LS) and maximum likelihood [39].

In contrast to the non-collaborative IPS, the computational architecture is more complex as the position estimate does not only depend on the device data, but also on the data gathered by nearby devices. Usually, the computational architecture of CIPS can be classified into two categories: centralized and decentralized. In a centralized architecture [72,78,82], the nodes/actors collect the unprocessed data from the sensors, which are sent to a central node that calculates the position estimate of all nodes. In a decentralized architecture [19,65,72,77,78,82], the role of nodes consists of acquiring and sharing (raw or processed) relevant data, but also in processing them in order to, for instance, self-determine their position. In both cases, the final position of a device is collaboratively estimated using the data and information provided by that and other devices.

**Figure 1:**
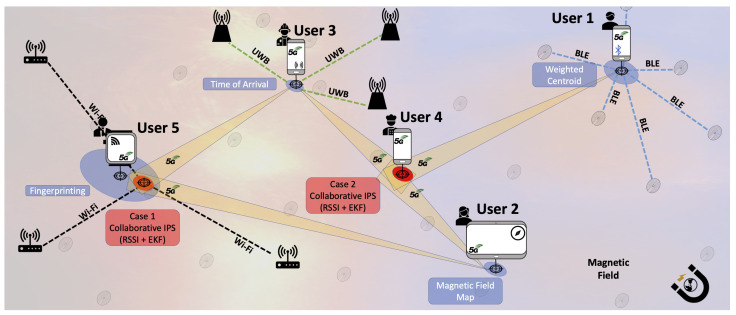
Representative example of a heterogeneous collaborative indoor positioning system. Source: Authors.

Figure 1 shows an illustrative example of a CIPS. First, notice the heterogeneity of this indoor positioning scenario, exhibited by all five users who use different approaches to self-estimate their position. As such, for the non-collaborative part: User 1 uses the BLE technology, RSSI technique, and the weighted centroid method; User 2 uses the Magnetic Field-based technology, Magnetic Field Map technique, and likelihood method; User 3 uses the UWB technology, ToA technique, and Multilateration method; and Users 4 and 5 use Wi-Fi technology, the fingerprinting technique, and *k*-NN method. The blue ellipses under the users represent the estimated position and its uncertainty. For the collaborative part, all users are using Device to Device (D2D) communications based on 5G technology. We present two cases where collaboration improved the results (see red ellipsoids).

- Case 1 aims to enhance the position accuracy of User 5, which has large uncertainty. The CIPS applies EKF to integrate the ranging information from Users 2 and 3 to estimate a better position.
- Case 2 aims to determine the position of User 4, who is not able to self-determine its position as it is far from the Wi-Fi area. The CIPS applies EKF to integrate the ranging information from Users 1–3 to estimate the position even if the non-collaborative part fails.

The main difference between CIPSs and traditional IPSs is that the CIPSs exploit the technologies of the systems both for communication between users and for distance estimation, and the methods consider not only individual information but that of the entire group of collaborators to estimate the position.

## 3. Research Methodology

This work introduces a systematic review on Collaborative Indoor Positioning Systems (CIPSs) based on the PRISMA guidelines [51]. Summarized, the review protocol is as follows. First, a set of research questions is formulated to establish the scope of the review. Then, a set of inclusion and exclusion criteria are defined, related to the stated research objectives and boundaries drawn from the research questions, in order to decide the relevance of every considered research article. Next, a rigorous study selection process is carried out, by first defining relevant search queries and running them against scientific digital libraries (Scopus and Web of Science in this work) to identify all potentially relevant studies. Subsequently, the found records are merged, duplicates removed, and screened against the inclusion and exclusion criteria in order to obtain the final set of relevant articles. These articles are then classified, and their features are extracted, mapped, and analyzed.

The research questions and inclusion and exclusion criteria used in this systematic methodology are described in Section 3.1 and Section 3.2, respectively. The study selection process is fully explained in Section 3.3, and the classification of studies is presented in Section 3.4.

### 3.1. Research Questions

The purpose of this systematic review is to assess and present an overview of research works in CIPSs, as well as present their results. In accordance with those goals, the following set of research questions was formulated:

**RQ1:** What are the infrastructures, architectures, technologies, techniques, and methods (also called algorithms) used in/for CIPSs?
**RQ2:** In which combination are technologies, techniques, and methods used in/for CIPSs?
**RQ3:** How have CIPSs been evaluated, and what are the metrics used?
**RQ4:** What are the limitations, current trends and gaps, and future research avenues that have been reported?

RQ1 specifies the overall goal of our review. Although infrastructures, architectures, technologies, techniques, and methods have been addressed in literature in the context of IPS, we focus here on their use in collaborative systems, and we draw parallels and differences with respect to non-collaborative systems. RQ2 specifically aims to gain an insight in the use of the different technologies, techniques, and methods in conjunction, as these form the core of CIPS. The objective of RQ3 is to present the evaluation metrics used, the type of evaluations performed in CIPS, and their distribution based on the data reported by authors. The goal of the RQ4 is to provide an overview of trends, gaps and limitations, and to provide the research community with avenues for future research in CIPS. Research questions are addressed in the sections indicated in Table 4.

### 3.2. Inclusion and Exclusion Criteria

The studies considered in this review are assessed based on the following inclusion and exclusion criteria.

#### 3.2.1. Inclusion Criteria

**IC1:** Any full, primary research article written in English and published in a peer-reviewed international journal or conference proceedings.
**IC2:** Any article that explicitly presents a Collaborative Indoor Positioning System for human use.

#### 3.2.2. Exclusion Criteria

**EC1:** Any articles that are not full papers (e.g., short papers, demo papers, extended abstracts), or are not primary research (e.g., reviews, surveys), or are not published in a peer-reviewed international conference or journal (e.g., white books, blog posts, workshop papers).
**EC2:** Any articles that do not propose or analyze as main topic at least one CIPS for providing a user’s indoor position (e.g., non-collaborative systems, outdoor systems, algorithms outside the context of a CIPS) or target non-human use (e.g., aerial drones, underwater robotic systems).
**EC3:** Any articles that do not consider the definition of collaboration as the action of joint working between neighboring actors to provide positioning (e.g., sensor fusion, data fusion algorithm, stand-alone device with multi-sensors cooperation).

### 3.3. Study Selection Process

**Figure 2:**
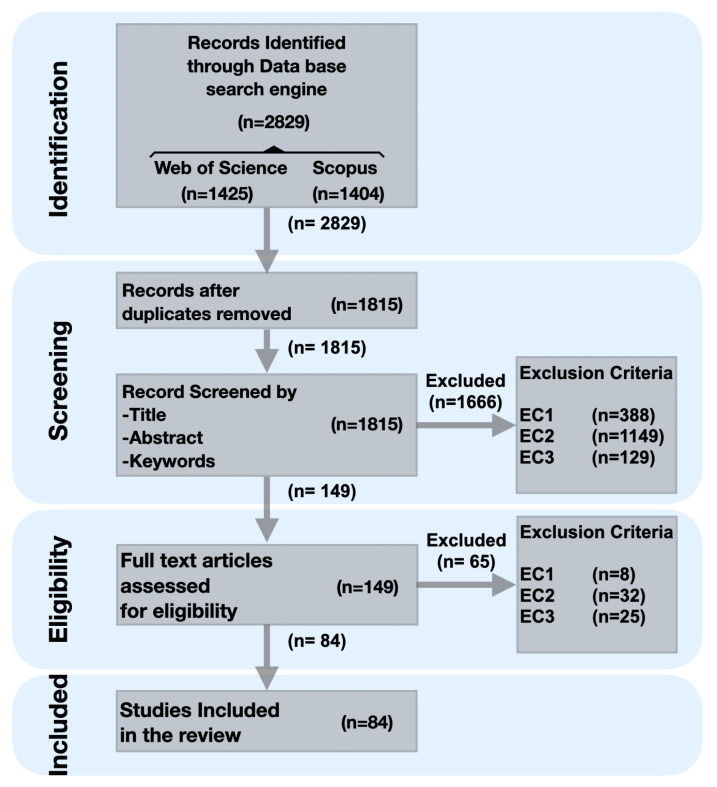
PRISMA flow diagram.

To select relevant articles, the PRISMA process for study selection was rigorously followed. First, to identify potentially relevant studies with respect to the research questions, an extensive article search was performed using the search engines from two curated scientific digital libraries, namely Scopus and Web of Science, during the identification phase. For each, an equivalent search query was specified, combining multiples keywords using boolean operators, in accordance with required syntax (see Appendix A.1). Subsequently, a screening process was carried out, by first removing duplicates, and subsequently screening the title, abstract, and keywords of the remaining articles against the inclusion/exclusion criteria. Finally, in the eligibility phase, the full remaining articles were checked against the eligibility criteria, to obtain a final set of included articles. The study selection process, with step-wise results, is schematically depicted using the PRISMA flow diagram in Figure 2. As a final set of eligible studies, 84 articles [40,41,42,43,44,45,46,47,48,49,50,63,77,79,80,81,83,84,85,86,87,88,89,90,91,92,93,94,95,96,97,98,99,100,101,102,103,104,105,106,107,108,109,110,111,112,113,114,115,116,117,118,119,120,121,122,123,124,125,126,127,128,129,130,131,132,133,134,135,136,137,138,139,140,141,142,143,144,145,146,147,148,149] were included in our review for full analysis.

### 3.4. Classification of the Studies

**Figure 3:**
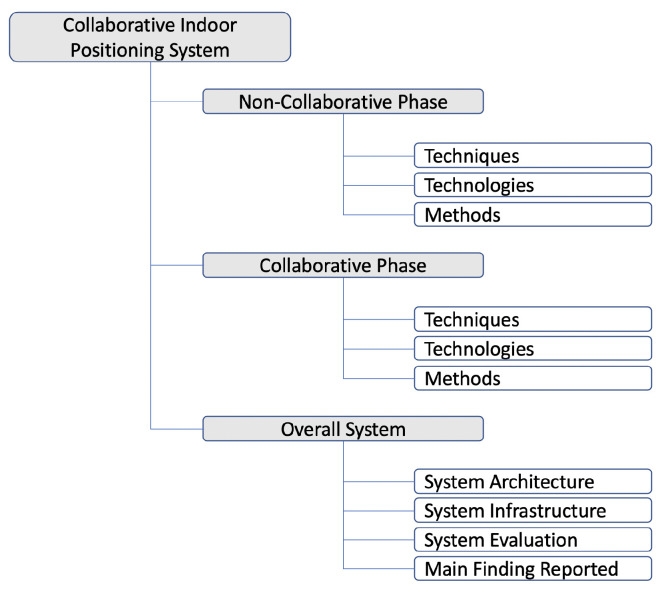
Structure of classification of studies.

Our classification scheme is driven by the typical logical breakdown of Collaborative Indoor Positioning Systems: (i) a non-collaborative phase, in which relevant data are acquired and, optionally, positioning is determined by every individual node; (ii) a collaborative phase, in which relevant data are exchanged between nodes, and positioning is determined based on exchanged data; (iii) an overall system, which coordinates and integrates the non-collaborative and collaborative parts of the system. In accordance, and considering the goals and research questions of our review, our classification scheme can be found in Figure 3 and is further elaborated in the next subsections.

#### 3.4.1. Non-Collaborative and Collaborative Phases

The non-collaborative and collaborative phases consider the technologies, techniques, and methods involved in the CIPS.

- **Technologies**. This category covers the technologies used to calculate the position on one hand (non-collaborative part) and to provide collaboration between users or nodes on the other hand. In one CIPS, the same or different technologies may be used for either part. Examples of technologies include IMU, Radio-Frequency Identification (RFID), and VLC for the non-collaborative part, and Bluetooth Wi-Fi and UWB for both parts.
- **Techniques**. Includes the techniques used for positioning and collaboration between users or nodes. Examples of techniques include fingerprinting, Dead Reckoning (DR), and Time of Arrival/Flight (ToA/ToF) for the non-collaborative part, and position sharing, Two-way Ranging (TWR), and Time Difference of Arrival (TDoA) for the collaborative part. We define techniques as the way certain technologies and derived data are organized and used to achieve positioning.
- **Methods**. Includes the algorithms and mathematical methods to compute the positioning and integrate collaboration among users. Examples of methods include Received Signal Strength (RSS)-based, PDR and *k*-NN for the non-collaborative part, and Particle Filter, Belief Propagation and EKF for the collaborative part. We define methods as a set of logical rules or processes to be followed in calculations in order to determine a positioning estimate.

#### 3.4.2. Overall System

The overall system considers the general features of the system and permits to classify the systems into four dimensions listed below.

- **System Architecture**. The System Architecture refers to the type of data processing architecture used in the CIPS, distributed or centralized in this review.
- **System Infrastructure**. The hardware deployed in the environment that the CIPS requires to operate such as BLE beacons, RFID tags, fixed cameras, and other ad hoc elements installed in the environment.
- **System Evaluation**. This category refers to how the system’s accuracy and performance are evaluated, for example, using numerical simulation, field tests, or both.
- **Main Finding(s) Reported**. In this category the main findings reported by the authors of the CIPS are classified. They are related with the evaluation metrics (position accuracy, position precision, system robustness, computational complexity, energy consumption), which are strongly linked with overarching concerns (i.e., concerns not specific to a particular architecture, infrastructure, technology, technique or method, but instead relevant for all systems), limitations of the systems and future research avenues.

The general organization of our classification scheme largely corresponds with those found in literature (discussed in Section 2.2, Section 2.3 and Section 2.4), yet differs in the fact that we do not attempt to group techniques, technologies, or methods. Instead, we exhaustively list all techniques, technologies, and methods encountered in our review in order to categorize papers according to their use of them.

## 4. Results

In this section, we present the results of the data analysis performed on the set of 84 articles identified during the article search and selection process, hereby focusing on research questions RQ1–RQ3. Particularly, the results include the distribution of the articles over time, the reported types of evaluation and evaluation metrics, the architectures and infrastructures, and the technologies, techniques, and methods, and the combination between the latter three. Additionally, a table that fully discloses all classification data for all papers in this review is available in Appendix A.2.

### 4.1. Evolution of CIPS over Time and Their Evaluation Metrics

**Figure 4:**
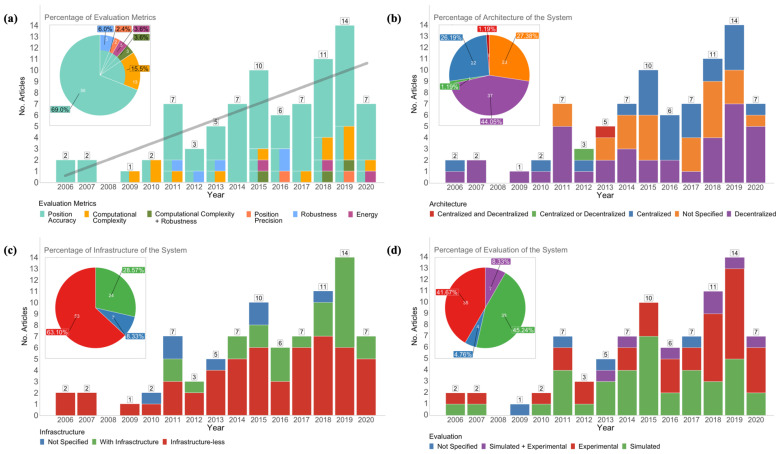
Evolution of the systems over time. (**a**) Distribution of the studies with their evaluation metrics. (**b**) Evolution of the systems’ architecture. (**c**) Evolution of the systems’ infrastructure. (**d**) Evolution of the systems’ evaluation. Source: Authors.

The stacked bar graph in Figure 4a shows the distribution of the 84 articles published regarding CIPSs, together with the reported evaluation metrics. A vertically split bar represents multiple evaluation metrics in a single article. For example, the bar of 2016 shows that all six articles of that year evaluated position accuracy, yet one article combined this with an evaluation of position precision, and two articles combined it with a robustness evaluation. Accumulated results are presented in the embedded pie chart.

Overall, the number of publications experienced a positive trend throughout the considered time period 2006–2020, as confirmed by the linear trend-line (light gray) calculated using the least-squares method. The first research was found in 2006, and in the first five years we observe an initially low number of publications. From 2010, we see a slow yet steady increase in the number of articles, with growth peaks in 2011, 2015, and 2019 that help to reach an average of approximately seven papers along this period. Additionally, it is clear that the most popular metric evaluation was the position accuracy (represented in cyan), which was present in all articles, mostly as a unique metric (69% accumulated) or reported in combination with others (31% accumulated). Of other metrics, a computational complexity evaluation was most often performed (15.5% + 3.6% in combination with robustness), followed by robustness (6% + 3.6%). Energy and position precision were least represented with 3.6% and 2.4%, respectively.

In the last four years, 46% (39 articles) of all research was done. Overall, no particular temporal trends in evaluation metrics can be discerned, although in the last four years we notice some increasing interest in computational complexity, some combined with robustness as evaluation metrics, but it is too soon (and the numbers are too low) to speak of a trend.

We note that the numbers reported for 2020 should be considered and analyzed with caution: (i) at the time of update (8 January 2021), the research databases may not yet include all the papers published in 2020; (ii) the COVID-19 health situation in 2020 was uncommon, with severe lock-downs worldwide. These restrictions impacted research, as many researchers were unable to attend their workplace for extended time periods and to perform empirical on-site experimentation; (iii) several relevant international venues for positioning and LBS were either canceled or postponed to 2021; (iv) some teams temporarily prioritized research on topics related to the health situation over their usual positioning and LBS work, such as contact-tracing. All these issues reduced the expected outputs (in terms of experiments and publications) for the year 2020; therefore, we cannot consider 2020 as representative in the overall evolution of works.

### 4.2. Infrastructure and Architecture

Figure 4b,c presents the results of the analysis towards the use of architectures and infrastructures in the 84 articles analyzed in this systematic review.

As can be observed from Figure 4b, two main architectures were encountered: decentralized, which is the most prevalent and accounts for 44.05% of articles, and centralized, which accounts for 26.19%. Remarkably, for 23 analyzed papers (27.38%), the architecture was not reported. Only two articles described a combination of the two above-mentioned architectures: one system reported a hybrid architecture that combined the centralized and decentralized approaches [100] (red in Figure 4b), and one system proposed an interchangeable architecture that could either operate as a centralized or decentralized system [96] (green in Figure 4b).

Figure 4b shows that from 2006 to 2014 the decentralized architecture was, in general, the most prevalent, with a ratio of 4:1. Subsequently, in the years 2015 to 2017, there was a surge of articles describing the use of a centralized architecture: the ratio between centralized and decentralized was 2.2:1. In the years 2018 and 2019, the distributed architecture presented a sharp increase in the number of articles, whereas the centralized decreased again: the ratio between centralized and decentralized swapped to 1:2.2. The CIPSs combining both were proposed in 2012 and 2013.

From Figure 4c it is clear that the majority of CIPSs (63.1%) were infrastructure-less (including 14 out of 53 systems based on Signals of Opportunity), versus 28.57% that need infrastructure. A few works, 8.33%, did not report whether or not infrastructure was used. Systems with infrastructure only slowly appeared since 2010 and generally increased in the last years. Nevertheless, for every year, the number of articles published regarding infrastructure-less systems outnumbered the number of articles regarding systems with infrastructure, with the exception of 2016 (equal amount) and 2019, in which we see infrastructure-based systems for the first time overtaking infrastructure-less systems (8 versus 6). It remains to be seen if this trends continues in the next years.

### 4.3. Non-Collaborative Technologies, Techniques, and Methods

**Figure 5:**
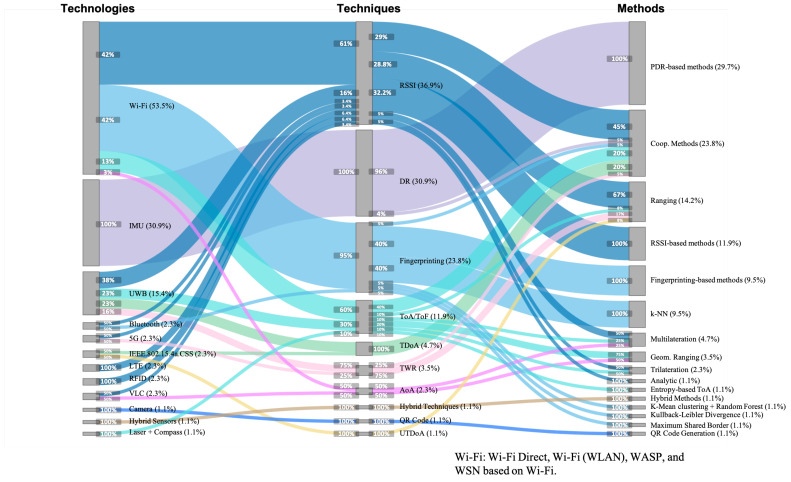
Non-collaborative technologies, techniques, and methods in CIPS. Source: Authors.

The Sankey diagram in Figure 5 shows the technologies (left), techniques (middle), and methods (right) for non-collaborative positioning estimation based on individually (non-collaboratively) acquired information. Articles were classified and grouped along these dimensions, and the groups were sorted in descending order. Each percentage (between brackets after the technology/technique/method name in Figure 5) denotes the number of articles (over the total set) in which a technology, technique, or method was used. Note that the sum of percentages within each dimension may transcend 100%, as the CIPS proposed in an article may use several technologies, techniques, or methods. A horizontal line denotes the combination of a technology with a technique (left in Figure 5) and the combination of a technique with a method (right in Figure 5) in a CIPS. The color of the lines is determined by the technique, as this best determines the technology and method used. It needs to be noted that some articles did not report the exact method used. Rather than classifying them as “unknown”, we classified these methods more informatively according to the technique they were used in combination with, using the suffix “-based”, i.e., PDR-based, RSSI-based, and Fingerprinting-based.

Figure 5 gives an overview of the frequency of use of each individual technology, technique, and method, but also on the combination of them. We observe that twelve different technologies have been used, of which Wi-Fi was predominant (53.5%). Inertial Measurement Unit (IMU) (30.9%) and Ultra-wide band (UWB) (15.4%) were also relatively well-studied, while the rest of the technologies received little attention: Bluetooth (2.3%), 5G (2.3%), IEEE.802.15.4a.CSS (2.3%), Long-Term Evolution (LTE) (2.3%), Radio-Frequency Identification (RFID) (2.3%), Visible Light Communication (VLC) (2.3%), Camera (1.1%), Hybrid Sensors (1.1%), and Laser + Compass (1.1%). Nine out of the twelve technologies appeared in just one or two papers. The Wi-Fi technology clusters the technologies Wi-Fi Direct, Wi-Fi (WLAN), Wireless Application Service Provider (WASP), and Wireless Sensor Network (WSN) based on Wi-Fi.

Moreover, we found ten different techniques. The four most representative techniques were Received Signal Strength Indicator (RSSI) (36.9%), Dead Reckoning (DR) (30.9%), Fingerprinting (23.8%), and Time of Arrival/Flight (ToA/ToF) (11.9%). Lesser represented techniques were Time Difference of Arrival (TDoA) (4.7%), Two-way Ranging (TWR) (3.5%), Angle of Arrival (AoA) (2.3%), Hybrid Techniques (1.1%), QR Code (1.1%), and Uplink Time-Difference-of-Arrival (UTDoA) (1.1%). Four out of the ten techniques appeared in just one or two papers.

Finally, sixteen different methods were encountered, with two clearly more studied than others: Pedestrian Dead Reckonings (PDRs) (29.7%) and cooperative methods (23.8%)—which involves those methods that are jointly used in both phases: the non-cooperative and cooperative phases. A second group of four methods was still reasonably well-studied: ranging (14.2%), Received Signal Strength Indicators (RSSIs)-based methods (11.9%), Fingerprinting-based methods (9.5%), and *k*-Nearest Neighborss (*k*-NNs) (9.5%). All other methods were studied less frequently: Multilateration (4.7%), Geometric Ranging (3.5%), Trilateration (2.3%), and with a 1.1% each the methods Analytic, Entropy-based Time of Arrival/Flights (ToA/ToFs), Hybrid Methods, *k*-Means Clustering + Random Forest, Kullback-Leibler Divergence, Maximum Shared Border, and QR Code Recognition. Almost half of the methods (7 out of 16) appeared in just one paper.

From the plot we can also derive that some works, no more than 14 (16.6%) to be exact, combined multiple positioning solutions in the non-collaborative part, as the sum of technologies, techniques, and methods was slightly higher than 100%. A further manual analysis revealed that eight of them (9.5%) combined IMU and Wi-Fi technologies [43,63,98,100,102,118,123,147]; two of them (2.4%) combined IMU with RFID [129] and UWB [144] technologies; one of them (1.1%) combined Wi-Fi and Bluetooth [142]; and three of them (3.6%) combined two different techniques based on Wi-Fi—AoA+ToA/ToF [50], RSSI+fingerprinting [42], and RSSI+ToA/ToF [133]).

The most interesting part of Figure 5 is the combination in which (non-collaborative) technologies, techniques, and methods were used in CIPS. Several observations stand out from the figure.

Regarding the technologies:

- The most used technology, Wi-Fi (used in 53.5% of all articles), was in the majority of cases combined with the Received Signal Strength Indicator (RSSI) technique (42% of articles using Wi-Fi technology), also to an equal amount with Fingerprinting (42%).
- The Inertial Measurement Unit (IMU) technology was exclusively used in combination with the Dead Reckoning (DR) techniques and the Pedestrian Dead Reckoning (PDR) methods (with the exception of a single use of a collaborative algorithm).
- Wi-Fi and Ultra-wide band (UWB) were the technologies that had been combined with the largest number of techniques (four techniques each). The most common technique for both technologies was Received Signal Strength Indicator (RSSI).

Regarding the techniques:

- Dead Reckoning (DR) was used in combination with a single technology, Inertial Measurement Unit (IMU).
- Received Signal Strength Indicator (RSSI) and Time of Arrival/Flight (ToA/ToF), respectively the first and fourth most used technique, were used in combination with the greatest number of different technologies (respectively seven and three technologies). The TDoA, TWR, and AoA techniques were used with two technologies each; all other techniques were used with a single technology (with the exception of the Fingerprinting that was used with Wi-Fi and Bluetooth).
- RSSI, Fingerprinting, and Time of Arrival/Flight (ToA/ToF) were used with the highest number of methods (six methods each).

Regarding methods:

- The two most popular methods, PDR-based and Cooperative methods, were used in almost half of the articles (53.5%). Together with the group of four reasonably well-used methods (i.e., Ranging, RSSI-based, Fingerprinting-based methods, and *k*-NN), they appeared in almost 98% of the reviewed papers. The remaining 10 methods were less common and appeared in less than 20% of papers.
- The popular Cooperative and Ranging methods (respectively second and third most used) were combined with a variety of techniques. Cooperative methods were used in combination with RSSI (45% inputs), TDoA (20% inputs), ToA/ToF (20% inputs), and with DR, TWR, Fingerprinting in 5% inputs each. Ranging methods were highly coupled with the RSSI technique (67% of inputs to the method), but it was also used with other techniques, namely TWR (17% inputs), ToA/ToF (8% inputs), and UTDoA (8% inputs). In contrast, *k*-NN exclusively worked with the Fingerprinting technique, which in turn was mainly used in combination with the Wi-Fi technology.
- Artificial Intelligence (AI) was present in three positioning methods, namely *k*-NN (in 9.5% of analysed works), and *k*-Means Clustering + Random Forest and Kullback-Leibler Divergence the (in 1.1% of analyzed works each).
- Almost half of the methods (7 out of 16) were only used in one article and were evidently each combined with a single technique and method.

### 4.4. Collaborative Technologies, Techniques, and Methods

**Figure 6:**
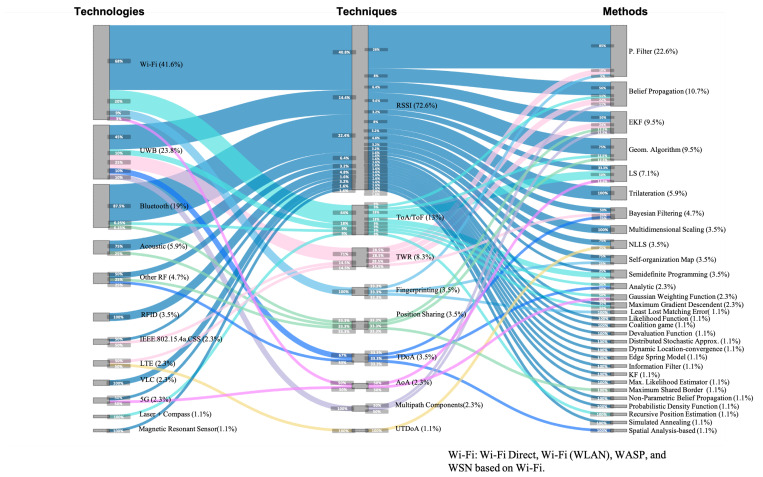
Collaborative technologies, techniques, and methods in CIPS. Source: Authors.

The Sankey diagram in Figure 6 shows the technologies, techniques, and methods used for the collaborative part of CIPS, where relevant (sensor) data are acquired and exchanged between actors/nodes, and collaborative positioning is calculated. It is constructed in the same way as for the non-collaborate part (see Section 4.3).

From Figure 6, we immediately notice a broader range of combinations considering the different technologies, techniques, and methods compared for the non-collaborative part of Collaborative Indoor Positioning Systems (CIPSs). A dominant technique, RSSI, arises from the diagram, yet it was combined with a multitude of technologies and methods.

We discerned twelve different technologies being used, of which Wi-Fi was used by almost half of the CIPSs (41.6%), followed by Ultra-wide band (UWB) (23.8%) and Bluetooth (19%). All other technologies were used in five or fewer papers each: Acoustic (5.9%), other RF technologies (4.7%), Radio-Frequency Identifications (RFIDs) (3.5%), IEEE.802.15.4a.CSS (2.3%), Long-Term Evolutions (LTEs) (2.3%), VLC (2.3%), 5G (2.3%), Laser+Compass (1.1%), and Magnetic Resonant Sensor (1.1%). Seven out of twelve technologies appeared in just one or two papers.

We encountered nine different techniques used in the collaborative phase, with an overwhelming majority of systems using Received Signal Strength Indicator (RSSI) (72.6%), followed distantly by Time of Arrival/Flight (ToA/ToF) (13%). All other technologies were only sporadically used: Two-way Ranging (TWR) (8.3%), Fingerprinting (3.5%), Positioning Sharing (3.5%), Time Difference of Arrival (TDoA) (3.5%), Angle of Arrival (AoA) (2.3%) Multi-path Components (2.3%), and Uplink Time-Difference-of-Arrival (UTDoA) (1.1%). Six out of nine techniques appeared in three or fewer papers.

With respect to methods, we found a large dispersion of 30 methods, with Particle Filter as the most popular (22.6%), followed by Belief Propagation (10.7%) Extended Kalman Filters (EKFs) (9.5%) and Geometric Algorithms (9.5%). The remaining 27 methods were applied in six or fewer works each: LS (7.1%), Trilateration (5.9%), and Bayesian Filtering (4.7%). Mutidimensional Scaling, Non-Linear Least Squares (NLLSs), Self-organizing Map, and Semidefinite Programming were 3.5% each. The Analytic, Gaussian Weighting Function and Max. Gradient Descendant were 2.3%. With 1.1% each, we had the following sixteen methods: Least Lost Matching Error, Likelihood Function, Coalitional Game, Devaluation Function, Distributed Stochastic Approx., Dynamic Location-convergence, Edge Spring Model, Information Filter, Kalman filter, Max. Likelihood Estimator, Max. Shared Border, Non-parametric Belief Propagation, Probabilistic Density Distribution, Recursive Position Estimation, Simulated Annealing, and Spatial Analysis-based.

The plot also shows that a few works, only nine (10.71%), combined multiple positioning solutions in the collaborative part, as the sum over technologies, techniques, and methods was slightly higher than 110%. A further manual analysis revealed that six papers combined two technologies using RSSI as technique (7.1%), which were Wi-Fi + acoustic [118], Bluetooth + acoustic [131], Wi-Fi + UWB [146], Bluetooth+Wi-Fi [42,43,142], and three papers combined two Wi-Fi techniques (3.5%), which were RSSI+ToA/ToF [133], RSSI + fingerprinting [102], and AoA+ToA/ToF [50]. In those nine papers, the technologies or techniques were fused on the collaborative positioning method.

Analyzing the combinations of technologies, techniques, and methods from Figure 6, we highlight the following results:

- The most used technology, Wi-Fi (used in 41.6% of all articles) was in the majority of cases combined with the RSSI technique (68% of articles using Wi-Fi technology), yet to a lower extent also with ToA/ToF (20%), Fingerprinting (9%) and minimally with AoA (3%).
- The top three technologies, Wi-Fi, UWB, and Bluetooth, were all combined with multiple techniques, respectively with four, five, and three. Specifically Wi-Fi with RSSI, ToA/ToF, Fingerprinting, and AoA; UWB with RSSI, ToA/ToF, TWR, TDoA, and Multipath components; Bluetooth with RSSI, ToA/ToF, and Positioning sharing, as can be observed in Figure 6.
- The RSSI technique was by far mostly used (72.6%) and was combined with a large variety of technologies and methods. It was mostly combined with the technologies Wi-Fi (40.8%), Bluetooth (22.4%), UWB (14.4%), Acoustic (6.4%), RFID (4.8%), Other RF (3.2%), VLC (3.2%), IEEE.802.15.4a.CSS (1.6%), Magnetic Resonant Sensor (1.6%), and it was combined with 24 of the 30 methods, with Particle Filter being the most used (28%) combination.
- From virtually every technique, there was a diversity of combined technologies and methods. Only Fingerprinting and Multipath Components were combined with a single technology, respectively Wi-Fi and UWB; all techniques, except UTDoA that appeared in just one paper, were combined with multiple methods.
- The most used method, Particle Filtering, was used 85% in combination with RSSI, 10% in combination with TWR, and 5% with Fingerprinting.
- Artificial Intelligence (AI) had a significant presence in collaborative methods, with more than 20 out of 30 methods. The most popular methods were Particle Filter, Belief Propagation, Least Square, and Bayesian Filtering, which were present in a 22.6%, 10.7%, 7.1%, and 4.7% of works, respectively. Other interesting AI methods were Mutidimensional Scaling, Non-Linear Least Squares (NLLSs), Self-organizing Map, and Semidefinite Programming, each present in 3.5% of works, followed by Gaussian Weighting Function and Max. Gradient Descendent with a presence in 2.3% papers each. The least common collaborative AI methods appeared in just one paper each and included Likelihood Function, Coalitional Game, Devaluation Function, Distributed Stochastic Approx., Information Filter, Max. Likelihood Estimator, Max. Shared Border, Non-parametric Belief Propagation, Probabilistic Density Distribution, Recursive Position Estimation, and Simulated Annealing.
- A majority of methods were only used once (16 of 30) or twice (6 of 30). Evidently, methods that were used once combined with a single technique and method.

### 4.5. Evaluation of Systems

Understanding how the systems were evaluated allows us to better interpret the significance of the results and qualify any comparison of reported results. Figure 4d presents the type of evaluation performed on the reported CIPSs. We discern experimental, simulated, both, or evaluation type not specified. Overall, the embedded pie chart illustrates a similar number of systems being simulated (45.24%) and experimentally (41.67%) evaluated, and a minority of systems both experimentally and simulated (8.33%) and not specified (4.76%). The bar chart reveals, over the years, a overall dominance of simulated evaluations until 2017. In the last three years (2018–2020) however, there was a large increase in experimental evaluations, combined with a relative drop in simulated evaluations, which caused the experimental to overtake the simulated evaluation (ratio roughly 2:1). Combined evaluations (i.e., both experimental and simulated) were only sporadically present over the time period 2006–2020.

## 5. Discussion

In this section, we further analyze and discuss the set of articles in light of the quantitative results presented in Section 4, trying to uncover the underlying reasons for the findings. We hereby deepen the answers to research questions RQ1–RQ3. Finally, based on this deeper analysis, we address research question RQ4 and point out limitations, gaps, and future research avenues.

### 5.1. Architectures and Infrastructure of Collaborative Indoor Position Systems

Regarding the architectures for CIPSs, centralized architectures are less used than decentralized ones. Articles reporting on centralized CIPSs often outlay implementation and deployment hurdles, which may deter their further use. Those problems include high complexity of the algorithms required to solve the positioning problem in a cooperative way [77,103,126,129], communication bottlenecks and delays because massive data exchange between nodes and centralized server [100,103], scalability in terms of the computational burden and concurrent users [95,100,129], and lack of robustness against failure [129]. In contrast, decentralized architectures are designed to share the computational processing among all the collaborative devices. Each node or actor pre-processes the collected data (for instance, calculating their position) and then broadcasts relevant information to other users. This procedure reduces the amount of transmitted raw data and alleviates the computation on the central node. The computational complexity metrics are predominantly performed for decentralized architectures in order to demonstrate their superiority in terms of computational optimization [77,90,109,131,132,137], whereas other performance metrics, such as the accuracy, are relegated to a second plane, as they depend primarily on technology, technique, and method used. In other words, decentralized systems offer the same positioning accuracy with fewer computational problems. This explains why a majority of systems preferred a decentralized architecture (44.05%) over a centralized one, especially in the last 4 years.

The choice between infrastructure and infrastructure-less approaches is related to the type of sensing technology in use. A majority of research focuses on positioning scenarios in existing buildings, some of them re-using already existing infrastructure (i.e., signals of opportunity). Typical scenarios that are receiving a growing interest involve locating people within houses, offices [63,115,123,131], and universities [40,43,63,79,98,101,119,120,128,131], where the viability of installing complex infrastructure is low in terms of costs, unlike industrial or warehouse scenarios, which are capable of developing and deploying robust and costly infrastructures designed for positioning. As a result, our review indeed shows that infrastructure-less approaches are predominantly selected for CIPSs in research (see Figure 4c), and that the majority of technologies used (see Figure 5 and Figure 6) are already present in the environment. They are either reused (i.e., Wi-Fi) or do not require any deployment (i.e., IMU, laser, compass).

### 5.2. Technologies, Techniques, and Methods in Collaborative Indoor Positioning Systems

#### 5.2.1. Analysis on the Non-Collaborative Part

The results provided in Section 4.3 showed that in 66 articles, positioning was done in two steps: each node or actor used an indoor positioning method (based on one or multiple positioning technologies) to get an initial estimation with only its collected data [40,41,42,43,44,45,46,47,48,49,50,63,77,79,80,81,83,84,86,87,89,91,92,93,94,97,98,100,101,102,103,104,106,107,108,109,110,111,113,114,116,117,118,119,120,123,124,125,127,128,129,130,131,132,134,135,136,137,141,143,144,145,146,147,150]. The estimated position or the raw data was later used in the collaborative part to enable or improve other user’s positioning. The remaining 18 articles were fully collaborative, as they relayed the position estimation of the nodes and/or actors completely to a collaborative method (i.e., denoted as “Cooperative methods” in Figure 5). In other words, stand-alone positioning was not performed in these systems, and the collected raw data were directly processed using the corresponding proposed collaborative method [85,88,90,95,96,99,105,112,115,121,122,126,133,138,139,140,142,148]. Therefore, the cooperative methods are excluded from the discussion in this section devoted to the non-collaborative part; for full details on the collaborative systems, please see Section 5.2.2. We also note that in two of these articles [133,142], authors proposed two different CIPSs.

Regarding stand-alone positioning in the non-collaborative phase, the most used methods were PDR [43,63,92,97,98,100,101,102,106,107,110,117,118,120,123,127,128,129,131,141,144,145,146,147], Ranging [42,46,47,81,93,109,111,113,130,132,144,150], RSS-based [40,41,43,48,77,84,89,103,123,129], *k*-NN [63,79,108,114,118,119,134,136], fingerprint-based [42,83,98,100,102,143,147,149], and multilateration [86,94,104,137] methods. Those methods were highly coupled to two main positioning techniques: RSSI (including fingerprinting techniques [108,110,114,116,134]) and DR, which in turn rely on communications technologies (mainly Wi-Fi) and inertial sensors respectively [40,41,42,43,45,48,50,63,79,81,83,84,85,87,88,89,90,93,95,98,100,102,103,104,105,108,109,112,114,116,118,119,122,123,129,130,133,134,136]. The major drawbacks of Wi-Fi and IMU as base positioning technologies are widely known. On the one hand, the positioning error provided by Wi-Fi-based positioning is around a few meters, whereas IMU-based solutions might suffer from accumulated drift errors. On the other hand, both can be considered infrastructure-less solutions. In the case of Wi-Fi, the already available network infrastructure for communications can be used for more-or-less accurate positioning (around a few meters) with no additional cost. As expected, most of collaborative works try to exploit widely used low-cost and simple IPSs with known issues, in order to improve them by means of collaboration. In fact, only a few CIPSs combined two or more positioning technologies in the non-collaborative [43,63,98,100,102,118,123,129] and in the collaborative [42,43,118,131] parts, although it is common in regular IPS. Just in one paper [43], multiple technologies—Wi-Fi+IMU in the non-collaborative part and Wi-Fi+Bluetooth in the collaborative part—were combined in both parts.

The positioning technologies that require large infrastructure, accurate calibration of the anchors, and provide high-accurate positioning have lower presence in the non-collaborative phase. The possible causes are (1) the less-likely integration of these technologies in wearable or human-tracking devices (e.g., smartphones have Wi-Fi support and inertial sensors, but only a few models support UWB); (2) the deployment costs might make it more attractive to explore infrastructure-less or less expensive solutions; (3) there is no need for collaboration as the deployed infrastructure offers full coverage of the operational area, and the positioning technology is accurate enough [19].

Another remarkable finding in the non-collaborative part is the lack of details of some key aspects of the CIPSs. Around a third of the reviewed papers did not provide enough details about the method used to provide the position estimate, only being cataloged as fingerprint-based [42,83,98,100,102,143,147,149], ranging [42,46,47,81,93,109,111,113,130,132,144,150] and RSS-based [40,41,43,48,77,84,89,103,123,129] methods. In those works, the authors considered the user’s positioning method in the non-collaborative part irrelevant, i.e., the main focus of the CIPS was to improve the user’s position in the collaborative part, regardless of the approach used in the non-collaborative part.

Table 5 presents a summary of the advantages and disadvantages of the five most popular non-collaborative methods, as well as their computational performance, positioning accuracy, and their implementation, among others. Regarding the PDR-based methods, although they provide a reasonable estimate of the trajectory, they accumulate positioning error over time. The ranging methods are negatively affected byNLOS conditions; however, in LOS conditions, they provide a good performance and distance estimation. RSSI-based methods are straightforward, and their positioning estimation accuracy relies on the quality of Radio Frequency (RF) signal strength measurements. The fingerprint-based methods need a good-quality radio map, i.e., a set of previous collected data/samples, to operate. Although *k*-NN is a fingerprint-based method with high accuracy and easy implementation, its computational complexity in the operational phase increases as the number of reference samples and APs increases.

#### 5.2.2. Analysis on Collaborative Part

The results on the collaborative part shows that the techniques based on RSSI were predominant, which in turn mainly used diverse communication technologies (i.e., radio frequency, sound and light). The RSSI was commonly used to estimate the distance between the emitter and receiver. In general, the term RSSI has been used as a synonym of ranging positioning technique [151,152,153]. Less frequent but still relevant techniques are based on ToA/ToF or Two-way Ranging (TWR), which are in most of cases also coupled to the communications technologies (Wi-Fi, Bluetooth, and UWB). It is important to mention that good results of estimating the position and performance in collaborative systems require a good interplay between technologies, techniques, and methods, so that their advantages excel and disadvantages are compensated. Considering the accuracy and precision positioning, the best CIPS are based on VLC [135] and UWB [115], which are technologies that already provide high accuracy in conventional positioning systems.

In the collaborative part, the CIPSs are mainly using methods based on RSSI (used as a synonym of ranging or RSSI ranging) to calculate the relative distance between the involved users and actors. However, in around half of the works, the authors proposed a specific method—not widely used by other researchers—for positioning in the collaborative part. Regarding those methods, we observed that they were proposed for different purposes. Particle filter, Gaussian Weight Function, and Multidimensional Scaling were implemented to enhance robustness in CIPSs [50,98,116,120]. Belief propagation is mainly used because of its potential to achieve high accuracy in collaborative position estimation, but at the expense of high computational complexity. Correspondingly, the Collaborative Indoor Positioning Systems (CIPSs) that use Belief propagation tend to balance the trade-offs between the computational complexity and the positioning accuracy [46,48,90,99,100,109,132,137]. Trilateration and Geometric algorithms have been mainly used in those CIPSs that attempt to improve the energy consumption [42,43]. The methods EKF and LS, in combination with the UWB technology, were used in [49,115] to improve the position precision.

As with the non-collaborative part, the most used collaborative methods present advantages and drawbacks. The six most used methods were Particle Filter [83,89,93,98,102,117,118,120,123,126,129,136,146,147,149,149]; Belief Propagation [46,47,96,109,130,131,132,135]; EKF [106,107,115,125,140,141,150]; Geometric Algorithm [43,124,127,134,142,143]; LS [45,49,133,137,148]; Trilateration [40,41,42,94]. One of the main advantages of the methods based on Particle Filter is their capability of handling non-Gaussian and non-linear estimations; however, their computational complexity increases (increment of the number of particles) as the position accuracy increases. Methods based on Belief Propagation exhibit high reliability and versatility to be used with different statistical models, yet they also incur a high computational cost. On the contrary, the EKF, Geometric Algorithms, LS, and Trilateration have as an advantage a low computational complexity. Although the EKF works with Non-linear models, it is only designed for Gaussian noise conditions. In Geometric Algorithm, the positioning accuracy is highly dependent on the location of the nodes. In case of a bad distribution of nodes, the positioning accuracy is negatively affected. Regarding the LS, one of its inconveniences is that it can only be applied to linear modes. The Trilateration method can only be applied if there are three or more non-collinear points, and its performance is extremely poor in NLOS conditions. Table 5 provides a summary of the advantages and disadvantages described above.

#### 5.2.3. Overarching Concerns

Sensor fusion is not usual in CIPS, neither in the non-collaborative nor in the collaborative parts, despite state-of-the-art IPSs combining multiple technologies to enhance their accuracy, robustness, and/or precision [154,155,156]. Only [43,63,98,102,118,123,129] applied sensor fusion in the non-collaborative part and [121] in the collaborative part. Similarly, none of the works considered a scenario where different non-collaborative positioning solutions (with different technologies, techniques, and/or methods) co-exist as shown in the exemplary scenario in Figure 1. In general, each CIPS has introduced a collaborative system that was built on top of a controlled and simple approach in the non-collaborative part. However, there are many alternatives to track and localize users in different environments. We consider the device—and therefore sensor—diversity should not be omitted, as real-world scenarios will encounter various heterogeneous data sources, and corresponding applications should be encouraged to consume data from as many sources as possible.

In addition to the aforementioned improvements, CIPSs present other advantages over the conventional IPSs approaches. Some Collaborative Indoor Positioning Systems (CIPSs) have extended the coverage without deploying additional expensive and/or complex infrastructure by using the users as auxiliary nodes [40,41,42]. Other Collaborative Indoor Positioning Systems (CIPSs) have reduced the positioning ambiguities—and therefore the positioning error—in harsh environments with NLOS by processing the absolute (non-collaborative) and relative (between users) positions with Belief propagation [46,47,135]. It seems that the indirect LOS provided by the users plays a key role to improve positioning.

Regarding the communication protocol and synchronization of the devices, the vast majority of the articles analyzed (90.5%) did not cover these aspects, as they mainly focused on demonstrating the effectiveness of the collaborative system rather than addressing practical problems arising in a real-world setting. The communication protocols identified in this review were User Datagram Protocol (UDP) [63] and Collection tree protocol (CTP) [94]. Several articles mentioned D2D communication, without specifying the exact protocol used (e.g., [79]). In order to address the synchronization problem between devices, TWR was the most used [47,79,126,128,129]. Alternatively, the Hop-synchronization with GNSS time was used as an accurate method to measure time of flight between Bluetooth nodes [45].

Rather surprisingly, some relevant overarching concerns were hardly or not addressed at all. For example, energy consumption was only considered in three articles, even though energy drain due to collaboration (when the positioning system runs as a background process), rather than for calculating own position, is highly relevant and may defer users from using a CIPS. Ref. [145] proposes an algorithm to save energy consumption reducing the re-broadcasting messages among users. Ref. [43] uses a decentralized architecture, measured the energy consuming of the main components of their CIPS, and they found the operating system (30%), Wi-Fi (20%), and Bluetooth module (14%) to be consuming the most. Both [42], using a centralized architecture, and [43] furthermore reached the same conclusion: scanning for devices (e.g., using bluetooth, BLE) or wireless AP (e.g., for Wi-Fi) is a critical energy-consuming component of a CIPS. Attempts to reduce the energy consumption, for example by interchanging continuous scanning by intermittent scanning, reduced position accuracy [43].

Moreover, even though privacy and security are addressed in traditional indoor positioning systems, these concerns were not discussed in CIPS literature—where focus is primarily on proof-of-concept to show improved accuracy. Nevertheless, CIPS are particularly vulnerable, as careless data exchange during the collaboration process (e.g., unencrypted communication, broadcasting raw sensor measurement, or calculated position estimates) may leave the user prone to third-party breaches and leak his/her position. Regarding privacy and security in non-collaborative indoor positioning systems-relevant for the non-collaborative part of CIPSs, we mention for example [157], which present a malicious check-in defense scheme based on the AP selection and Big data analysis [158], which introduces a practical privacy-preserving indoor localization using outsourcing scheme and a security analysis [159,160], which discuss solutions based on *k*-NN + Paillier cryptosystem, Support Vector Machine (SVM) and k-anonymity, among others.

### 5.3. Evaluation of Collaborative Indoor Position Systems

One of the main pillars of CIPSs evaluation is how the experiments are designed and planned. Even though experimental evaluations are preferred, and there was a large increase in experimental evaluation during 2018, 2019, and 2020 (overtaking the amount of simulation-based evaluation), overall, we still observe a slightly higher number of simulation-based evaluations (i.e., 45.24% simulations versus 41.67% empirical experiments). Experimental evaluations best mimic circumstances and operational conditions of a real-life scenarios [43,79], yet they are more difficult to set up and perform [63,129], time-consuming [44,117,131], prone to various types of failure and errors [43,129], are and (potentially) costly [115,125,129]. Additional difficulties of experimental evaluations are the difficult-to-control practical issues faced in real-world scenarios, for example, the loss of precision due to signal attenuation/interference [43,121] and NLOS conditions [43].

Simulations provide a controlled environment, where both data and collaborative algorithms can be simulated. This eliminates hardware failures and allows researchers to easily perform different runs of an experiment with different configurations. For example, the number of users [130], quantity and density of the reference points [46,130], (simulated) hardware configuration in the environment [99,126,133], and even the environment itself [46,77] can be easily modified in different runs of the experiment. Balancing the two, a minority of articles (8.33%) presented a mixed evaluation, whereby the experimental part corresponded to simplified tests in real environments to validate the system, and simulations were used to test it in more complex environments [63,100,118].

Regarding the metrics, all the reviewed works included the positioning accuracy, as we can observe in Figure 4d. Going deeper in this particular evaluation metric, we observe that different measures have been provided which, in descending order, are as follows: the Cumulative distribution Function (CDF) of the positioning error [43,44,79,80,108,112,113,116,118,119,126,129,131,134,150], the Root Mean Square Error (RMSE) [48,49,77,109,121,122,126,135], the standard deviation of the error [40,41,42,63,80,110], the minimum mean square error [46,117,129,130,133], and finally the average positioning error [105,123,132,134]. This makes comparison of accuracy unfeasible.

Computational complexity evaluates the performance of a system considering the following aspects: the workload required to estimate the position collaboratively [46,77,122,130], the communication overhead [109], and the execution time to solve the positioning problem [46,132]. Some of the suggested approaches to reduce the computational complexity are (i) apply the collaborative positioning algorithm to a restricted set of users to reduce execution time [46,132]; (ii) formulate the problem of collaborative positioning as a quasi-convex feasibility problem to deal with the complexity of the non-convex structure models, which permits to reduce the computational load [77]; (iii) use a parametric belief propagation scheme and an analytical approximation to compute peer-to-peer messages in order to reduce the communication and computational cost [109,130].

The robustness determines how invariable a positioning system is under variations on the input data or execution failures. Just a few CIPSs were proposed with the main aim of increasing their robustness [47,50,98,116,120]. The algorithms used to measure robustness are against the ranging error, limited number of online samples or peer users, outdated fingerprinting database, and node failure. Some strategies to provide robustness are (i) to use a sum product algorithm over wireless networks to provide robustness again nodes failure [47]; (ii) to use a Gaussian neighborhood weighting method to eliminating multiple-bounce reflection paths [50]; (iii) to use a Multidimensional scaling and Procrustes analysis to exhibit robust performance in cases with a limited number of online samples or peer users, large ranging errors, and fluctuated RSS readings [116].

The two least represented evaluation metrics, energy consumption [42,43,144] (discussed in Section 5.2.3) and position precision [49,115] (both based on UWB technology), have only recently been considered.

### 5.4. Recommendations, Gaps, and Limitations

On the basis of the results and analysis performed in this systematic review, i.e., the current state of the art, we present the following recommendations for researchers regarding the development of collaborative indoor position systems for positioning of humans:

1. Architecture: A decentralized architecture is the most suitable option for a collaborative approach since it avoids communication bottlenecks, delays in response times, and dependence on a server. However, computing algorithms on (restricted) user devices limits the implementation of complex algorithms and, due to device variability, its performance might not be homogeneous for all users.
2. Infrastructure: A CIPS based on Infrastructure-less approach or based on signals of opportunity might be preferable, due to the continuous mobility of users in different environments, and the cost of developing an infrastructure to provide coverage of the operational area. In addition, an Infrastructure-less approach provides versatility to the system in order to be used in a larger number of scenarios. However, the lack of an ad hoc infrastructure for the CIPS implies a challenge in its design in order to compensate for the inaccurate positioning that the uncontrolled environments provide. Only for specific real-world scenarios, an infrastructure-based approach may be preferable.
3. Technologies: Despite the great accuracy and precision positioning provided by some technologies (mainly VLC, UWB, and 5G), Wi-Fi and BLE might currently be better suited, as other relevant factors are the ubiquity of the technologies used, the low implementation costs, and the low energy consumption that Wi-Fi and BLE offer. An evolution in general availability and supporting hardware, e.g., particularly in the case of 5G, may cause a shift in preferred technology.
4. Techniques: From the point of view of positioning accuracy and considering Wi-Fi as main positioning technology, Wi-Fi Fingerprinting is widely used because the position of the anchors (APs) is not needed. However, the techniques based on RSSI perform better as the geometry and distribution of the APs are well known. Further investigation of the supporting infrastructure—e.g., estimating the APs by manual inspection or automatic detection [161,162]—might allow the replacement of fingerprint-based with more accurate RSSI-based methods.
5. Methods: Due to the diversity of scenarios and conditions in which the systems have been tested, it is difficult to specify which method is the most appropriate. We consider that different alternative methods should be compared in different dimensions—mainly accuracy, precision, robustness, and computational cost—when a new CIPS is proposed, and the final proposed one should be selected according to some pre-defined criteria (e.g., best positioning error, lowest execution time, or a trade-off between the two).

The above recommendations may serve as a guide to follow when designing further CIPS. However, as the wide variety of solutions reported in the reviewed papers shows, the decisions on each part must be tailored to the specific needs of each system.

The analysis of the reviewed papers also highlighted some restrictions and/or limitations, which can be considered gaps in current research and provide future research opportunities.

- The proposed CIPSs tend to focus on excelling in one relevant characteristic, mainly the deployment costs, the computational complexity, the real-time operation, energy consumption, or the positioning accuracy. The main limitation of current CIPSs is that none of them try to balance all these aspects, specially in complex environments.
- In general, the CIPS select a single technology for the non-collaborative part and a single technology for the collaborative part. Generally, the reviewed CIPS neither exploit sensor fusion nor multiple positioning alternatives. We consider that technology diversity in both parts might make the CIPS more robust, as it has been demonstrated in conventional IPS.
- None of the reviewed works = considered the privacy of the users nor the security of the CIPSs. Privacy is a main overarching concern that has already been regulated in many countries (e.g., the European General Data Protection Regulation (GDPR) [163]). The vast majority of positioning solutions (in the non-collaborative and collaborative phase) rely on communication technologies that can be attacked (i.e., jamming or spoofing mainly) to alter the outputs of the positioning system and/or the sensing data processed by the user, which might be considered a security breach of the CIPS. Energy consumption is also a relevant overarching concern, which may deter users from using a CIPS, and this area is insufficiently studied.
- The evaluations of the CIPSs are tightly coupled to the technology used in the non-collaborative part. The community needs an evaluation framework able to objectively evaluate the collaborative part of the CIPS with independence to the positioning technology used in the non-collaborative part. An important part of such a framework is comparable evaluation metrics. Moreover, evaluation considering multiple technologies working simultaneously has not been widely explored yet.
- Evaluation is done over simulations in almost half of reviewed works because it does not require deploying expensive hardware and manual labor. Although some simulated environments are able to mimic the real world, a comprehensive empirical evaluation is needed to demonstrate the feasibility of the proposed CIPSs in realistic conditions. A repository of extensive multi-sensor and multi-user datasets for that purpose could enhance research reproducibility, enable the fair comparison of CIPSs, reduce evaluation costs (assuming the datasets are publicly available), and be an incentive to further research CIPSs.

## 6. Conclusions

This article presented a systematic review on CIPSs for humans. After a well-defined search phase, 84 relevant articles were identified in the time frame 2006–2020, which were subsequently classified along the following dimensions: architecture, infrastructure, technologies, techniques, methods, and evaluation metrics. The performed analysis demonstrated the growing interest within the scientific community for the study of the CIPSs, with an overall increasing number of articles over the years.

Our study shows a predominant use of a decentralized architecture, with an increase especially in the last 3 years. Cited disadvantages for centralized architectures include computational complexity, communication bottlenecks, scalability, and lack of robustness against failure. Regarding infrastructure, our study revealed a large dominance of infrastructure-less systems, which seem to be related to practical issues rather than technical ones, including use of already available hardware and an overall lower cost, which are more suitable for common scenarios. This does not take away the possibility of an infrastructure-based solution, as such solutions, while requiring more effort and being more expensive, have the potential to yield more accurate results than the infrastructure-less ones.

Regarding technologies, techniques, and methods, we separately analyzed the collaborative and non-collaborative parts of CIPSs. With respect to the non-collaborative part, in which relevant data are acquired and (optionally) positioning is determined by every individual node, the results show a wide diversity of technologies, techniques, and methods, making it difficult to declare a winning combination. Wi-Fi/RSSI, Wi-Fi/fingerprinting, and IMU/Dead Reckoning (DR) are widely used and are among the preferred combinations of technology and techniques in the literature for the non-collaborative part. They are recommended in scenarios where deployment costs need to be low (they are infrastructure-less or depend on signals-of-opportunity), and the system needs to work/be implemented on smartphones. We consider that fingerprint-based methods could be improved with additional knowledge of the environment, replacing them with more accurate RSSI-based methods. In contrast, for the collaborative part, in which relevant data are exchanged between nodes and positioning is determined based on exchanged data, RSSI based on Wi-Fi and Bluetooth technologies are popular among researchers due to their ubiquity, total infrastructure-less nature, low energy usage, and low cost. Regarding the method, none of them stood out, as they have different objectives. For instance, the Belief propagation provides high accuracy at the expense of high computational costs.

Some CIPS are fully collaborative, and the non-collaborative part just gathers data, which are later processed by a collaborative method. Our recommendations are for decentralized systems where the non-collaborative and collaborative parts can work independently to provide positioning to the users. In the centralized collaborative methods, if the central node fails, the position estimation cannot be estimated for any user.

So far, most evaluations of CIPSs in the literature have relied on simulations. However, in recent years there has been a growing interest in experimental evaluations, so in the short term we may be looking at a turnaround. Empirical/experimental evaluation better mimics complex real-world conditions, and the obtained results are more relevant for the community than the simulation-based results. However, the relevant results are obtained at the expense of manual labor and, sometimes, expensive hardware deployments. We consider that creating a repository of heterogeneous datasets, as done in regular IPSs, is necessary for multi-scenario cost-less evaluation.

CIPSs have demonstrated several benefits over non-collaborative IPSs. They may expand the coverage area of indoor location systems, e.g., by providing positioning to users located in areas uncovered by infrastructure. In addition, they reduce the positioning error by including the information of other users within the algorithms and by adding more reference points to be used to compute the position of a group of users. However, the CIPSs have disadvantages such as the computing time, the large computational processes of all nodes, and energy consumption. On this basis, one of the most important trade-offs and gaps found in literature is related to balancing the positioning accuracy, the real-time restrictions, and computational complexity of the method with respect to improving the energy efficiency.

We consider that there is still ample opportunity for improvement and further research in the area of collaborative positioning. As most promising future avenues, we see exploiting sensor fusion at the non-collaborative and collaborative parts; considering device and technology diversity in the CIPS architecture; enhancing the security and privacy of the positioning systems and LBS; and defining a more comprehensive evaluation setup that considers multiple realistic scenarios (either through empirical experiments and/or open-available datasets).

## Figures and Tables

**Table 1:** Different indoor positioning technologies classification reported.

Gu et al. [55] (2009)	Mautz [19] (2012)	Basiri et al. [14] (2017)	Mendoza-Silva et al. [53] (2019)
Infrared Vision-based Magnetic Audible Sound Ultrasound Radio Frequency	Infrared Camera Magnetic Localization Sound INS UWB WLAN/WiFi RFID Tactile and Combined Polar Systems High sensitive GNSS/Assisted GNSS Pseudolite Infrastructure systems Other RF (Cellular Networks, Zigbee, Radar, DECT Phones, Digital TV, FM radio)	Infrared Market or reflective element Infrared Light image feature matching Light image feature matching Light image market Magnetometer Sound UWB ToF WiFi RSS WiFi ToF/AoA RFID active Bluetooth RSS Tactile on user device Tactile Environment Tactile Odometer Pseudolite GNSS Electromagnetic Systems Barometer Mobile Network	Light Computer Vision Magnetic Field Sound Dead Reckoning UWB WiFi RFID and NFC Tactile Odometer BLE Other Technologies (Cellular Network, ZigBee, 5G)

**Table 2:** Different indoor positioning technique classifications reported.

Liu et al. [52] (2007)	Gu et al. [55] (2009)	Zafari et al. [54] (2019)	Mendoza-Silva et al. [53] (2019)
Triangulation ‒ Lateration * ToA * TDoA * RSS-based * RToF * PoA ‒ Angulation-AoA Scene Analysis Proximity	Triangulation ‒ RSS ‒ ToA ‒ AoA Fingerprinting Proximity Location Vision Analysis	RSSI Channel State Information Fingerprinting/Scene Analysis AoA ToA TDoA RToF PoA	AoA ToA TDoA RSS

**Table 3:** Different indoor positioning method classifications reported.

Güvenc and Chong [57] (2009)		He and Chan [56] (2016)	Yassin et al. [72] (2017)	Chen et al. [66] (2017)
In LOS scenarios Maximum likelihood (ML) ‒ ML ‒ Two-Step ML ‒ Approximate ML Least Squares (LS) ‒ Non-Linear LS ‒ Linear LS through Taylor’s Series Expansion ‒ Contrained Weighted LS	In NLOS scenarios Maximum likelihood (ML) ‒ ML utilizing NLOS Statistic ‒ Identification & Discard based ML Least Squares (LS) ‒ Weighted LS ‒ Residual Weighting Constrained Localization ‒ Constrained LS with Quadratic Programming ‒ Constrained LS with Linear Programming ‒ Geometric Constrained Localization ‒ Interior Point Optimization Robust estimator ‒ M-estimator ‒ Least Median of Squares	Deterministic ‒ Euclidean Distance ‒ Cosine Similarity ‒ Tanimoto Similarity ‒ K Nearest Neighbors ‒ Support Vector Machine ‒ Linear discrimination Analysis Probabilistic ‒ Maximum Likelihood ‒ Bayesian Network ‒ Expectation-Maximization ‒ Kullback-Leibler Divergence ‒ Gaussian Process ‒ Conditional Random Field	Triangulation-based Scene Analysis-based Proximity-based	Geometric-based Fingerprint-based

**Table 4:** Research questions and sections.

Research Question	Systematic Review Section
Research Question 1	Results, Section 4.2 and Section 4.4
	Discussion, Section 5.1 and Section 5.2
Research Question 2	Results, Section 4.4
	Discussion, Section 5.2
Research Question 3	Results, Section 4.5 and Section 4.1
	Discussion, Section 5.3
Research Question 4	Discussion, Section 5.4
	Conclusion, Section 6

**Table 5:** Summary of advantages and disadvantages of the most used non-collaborative and collaborative methods. The top section presents the five most used non-collaborative methods. The bottom section presents the six most used collaborative methods.

	Method	Advantages	Disadvantages
Non-Collaborative	PDR-based	Provides reasonable estimate of a walking person’s trajectory moving in a steady way	Suffers from accumulative errors
	Ranging	Presents good performance on LOS when the signal propagation is well modeled (e.g., VLC technology and Lambertian radiation pattern)	Relies on radio wave propagation model Geometry of the scenarios may affect the estimation accuracy NLOS condition usually degrades the distance estimation
	RSSI-based	very simple implementation Low computational cost	Depends on radio propagation model characterization Accuracy of the position estimation directly dependent on the RF signal strength quality
	Fingerprint-based	Uses empirical data for calibration and operation, which might mimic better the real scenario	Data collection can be very demanding Radio map’s quality degrades if environmental conditions change
	*k*-NN	Widely known and very simple implementation Low computational cost and high accuracy	Selection of K determines the performance It is a universal classification/regression model. Therefore, it does not consider the logarithmic nature of the RSS values and the non-linear nature of the signal propagation. Very demanding if the number of reference samples and number of APs are both high.
Collaborative	P. Filter	Capable of handling non-Gaussian and non-linear estimations Methodologically simple and flexible Permits to control the effects of increasing the number of dimensions of the state space Able to approximate any probability density function in the state space	Performance degrades considerably as the state-space dimension increases (curse of dimensionality) Number of particles is a trade-off between computational complexity and accuracy Issue of filter initialization
	Belief Propagation	High reliability Works with a wide variety of statistical models Efficient computing of distribution based on the graphical model Easy representation of multi-modal distributions	The computational costs are considerably high
	EKF	Capable of handling Non-linear models Low computational complexity	EKF is designed for Gaussian noises
	Geom. Algorithm	Low computational complexity, due to estimate the position using only the geometry based on the signal parameters	Accuracy is directly related to the geometric positioning of the nodes
	LS	Easy to implement Provides a solution of relatively low complexity	Works only with linear models
	Trilateration	Low computational complexity and easy implementation with basic geometry principles	3 non-collinear points are needed Requires LOS measurement

## Data Availability

Not applicable.

## Abbreviations

The following abbreviations are used in this manuscript:

**AAL**	Ambient Assisted Living
**AI**	Artificial Intelligence
**AoA**	Angle of Arrival
**AP**	Access Point
**BLE**	Bluetooth Low Energy
**CDF**	Cumulative distribution Function
**CIPS**	Collaborative Indoor Positioning System
**CSI**	Channel State Information
**CTP**	Collection tree protocol
**D2D**	Device to Device
**DR**	Dead Reckoning
**EKF**	Extended Kalman Filter
**FM**	Frequency Modulation
**GLONASS**	Globalnaya Navigazionnaya Sputnikovaya Sistema
**GDPR**	General Data Protection Regulation
**GNSS**	Global Navigation Satellite System
**GPS**	Global Positioning System
**IMU**	Inertial Measurement Unit
**IoT**	Internet of Things
**IPS**	Indoor Position System
***k*-NN**	*k*-Nearest Neighbors
**LBS**	location-based service
**LOS**	Line-of-sight
**LS**	Least Square
**LTE**	Long-Term Evolution
**MEMS**	Microelectro-Mechanical System
**NLLS**	Non-Linear Least Square
**NLOS**	Non-line-of-sight
**PDR**	Pedestrian Dead Reckoning
**PRISMA**	Preferred Reporting Items for Systematic Reviews and Meta-Analyses
**RF**	Radio Frequency
**RFID**	Radio-Frequency Identification
**RMSE**	Root Mean Square Error
**RSS**	Received Signal Strength
**RSSI**	Received Signal Strength Indicator
**SVM**	Support Vector Machine
**TDoA**	Time Difference of Arrival
**ToA**	Time of Arrival
**ToA/ToF**	Time of Arrival/Flight
**TWR**	Two-way Ranging
**UDP**	User Datagram Protocol
**UTDoA**	Uplink Time-Difference-of-Arrival
**UWB**	Ultra-wide band
**VLC**	Visible Light Communication
**WASP**	Wireless Application Service Provider
**Wi-Fi**	IEEE 802.11Wireless LAN
**WSN**	Wireless Sensor Network

## Appendix A

### Appendix A.1. Search Queries

Table A1 presents the search queries used to retrieve information from the database of Scopus and Web of Science on 8 January 2021. The search encompassed the period from 2006 until 2020.

**Table A1:** Scopus and Web of Science search queries.

Database	Input Query	No. Articles
Scopus	(TITLE-ABS-KEY (((Collabora* OR Coopera*) AND Indoor) AND (Position* OR Track* OR Locati* OR Locali* OR Navigat*)) AND LANGUAGE (english))	1404
Web of Science	TS=((Collabora* OR Coopera*) AND Indoor AND (Position* OR Track* OR Locati* OR Locali* OR Navigat*))	1425

### Appendix A.2. Articles Included in the Systematic Review

Table A2 fully discloses all classification data for all papers included in this review.

**Table A2:** Information of the 84 articles included in the systematic review grouped by publication year. It includes the technology, technique, and method for the non-collaborative and collaborative parts. Arch stands for system architecture, which can be centralized (C), decentralized (D), centralized and decentralized (C&D), centralized or decentralized (C/D), or not specified (N/S). Infr stands for system infrastructure, which can be with infrastructure (W/I), infrastructure-less (I-L), or not specified (N/S). Eval stands for system evaluation and can be simulated (S), experimental (E), simulated+experimental (S+E), or not specified (N/S). Eval Metric stands for evaluation metrics and can be position accuracy (PA), position accuracy+robustness (PA+R), position accuracy+computational complexity (PA+CC), position accuracy+energy (PA+E), position accuracy+computational complexity+robustness (PA+CC+R), or position accuracy+position precision (PA+PP).

Year	Ref.	Technology		Technique		Method		Arch.	Infr.	Eval.	Eval. Metric
		Non-Collaborative	Collaborative	Non-Collaborative	Collaborative	Non-Collaborative	Collaborative				
2020	[143]	Bluetooth	Bluetooth	F. printing	RSSI	F. printing-B.	Geom. Algorithm	D	I-L	E	PA
	[144]	IMU, UWB	UWB	DR, RSSI	RSSI	PDR-B. M., Ranging	Bayessian F.	D	W/I	S	PA+E
	[145]	IMU	Bluetooth	DR	RSSI	PDR-B. M.	EKF	D	I-L	E	PA
	[146]	IMU	Wi-Fi, UWB	DR	RSSI	PDR-B. M.	P. Filter	D	I-L	S+E	PA+CC
	[147]	IMU, Wi-Fi	UWB	DR, F. printing	TWR	PDR-B. M., F. printing-B	P.Filter	D	I-L	E	PA
	[148]	5G	5G	RSSI	RSSI	Coop. Algorithm	LS	N/S	W/I	S	PA
	[149]	Wi-Fi	Bluetooth	F. printing	RSSI	F. printing-B	P. Filter	C	I-L	E	PA
2019	[141]	IMU	UWB	DR	TWR	PDR-B. M.	EKF	D	W/I	E	PA
	[140]	UWB	UWB	TWR	TWR	Coop. Algorithm	EKF	C	W/I	E	PA
	[139]	UWB	UWB	TDoA	TDoA	Coop. Algorithm	Bayesian F.	N/S	W/I	E	PA
	[138]	UWB	UWB	TDoA	TDoA	Coop. Algorithm	Analytic	N/S	W/I	E	PA
	[137]	5G	5G	AoA	AoA	Multilateration	LS	D	I-L	S	PA+CC
	[49]	UWB	UWB	ToA/ToF	ToA/ToF	Entropy-based ToA	LS	D	I-L	S	PA+PP
	[81]	Wi-Fi	Wi-Fi	RSSI	RSSI	Ranging	Multidimensional Scaling	N/S	I-L	S	PA+CC+R
	[77]	VLC	VLC	RSSI	RSSI	RSSI-B. M.	Max. Likelihood E.	D	W/I	S	PA+CC
	[80]	RFID	RFID	RSSI	RSSI	Anlytic	Multidimensional Scaling	C	W/I	E	PA
	[135]	VLC	VLC	RSSI	RSSI	Trilateration	B. Propagation	D	W/I	E	PA
	[79]	Wi-Fi	Wi-Fi	F. printing	ToA/ToF	KNN	Analytic	D	I-L	E	PA
	[137]	Wi-Fi	Wi-Fi	F. printing	RSSI	KNN	Geom. Algorithm	N/S	I-L	E	PA
	[46]	UWB	UWB	RSSI	RSSI	Ranging	B. Propagation	D	I-L	S	PA+CC
	[142]	Wi-Fi, Bluetooth	Wi-Fi, Bluetooth	RSSI	RSSI	Coop. Algorithm	Geom. Algorithm	C	W/I	S+E	PA
2018	[134]	Wi-Fi	Wi-Fi	F. printing	RSSI	KNN	Geom. Algorithm	N/S	I-L	E	PA
	[48]	Wi-Fi	Other RF	RSSI	TDoA	RSSI-B. M.	Spatial Analysis-based	N/S	I-L	S	PA
	[129]	IMU, RFID	RFID	DR, RSSI	RSSI	PDR-B. M., RSSI-B. M.	P. Filter	C	W/I	E	PA
	[133]	Wi-Fi	Wi-Fi	RSSI, ToA/ToF	RSSI, ToA/ToF	Coop. Algorithm	LS	N/S	N/S	E	PA
	[132]	UWB	UWB	TWR	TWoA	Ranging	B. Propagation	N/S	W/I	S	PA+CC
	[43]	IMU, Wi-Fi	Wi-Fi, Bluetooth	DR, RSSI	RSSI	PDR-B. M.	Geom. Algorithm	D	I-L	E	PA+E
	[131]	IMU	Bluetooth, Acoustic	DR	RSSI	PDR-B. M.	B. Propagation	D	I-L	E	PA+CC+R
	[130]	Wi-Fi	Wi-Fi	RSSI	RSSI	Ranging	B. Propagation	N/S	I-L	S	PA+CC
	[44]	Wi-Fi	Bluetooth	F. printing	RSSI	K-mean clustering+R. Forest	P. Filter	C	W/I	E	PA
	[128]	IMU	UWB	DR	TWR	PDR-B. M.	Bayesian F.	D	I-L	S+E	PA
	[63]	IMU, Wi-Fi	Wi-Fi	DR, F. printing	F. printing	PDR-B. M., KNN	Least Lost Matching E	D	I-L	S+E	PA
2017	[127]	IMU	Other RF	DR	Pos. Sharing	PDR-B. M.	Geom. Algortihm	C	W/I	S	PA
	[126]	LTE	LTE	TDoA	TWR	Coop. Algorithm	P. Filter	D	I-L	S	PA
	[125]	Hybrid S.	Other RF	Hybrid Techni.	RSSI	Hybrid Methods	EKF	N/S	I-L	N/S	PA
	[124]	Laser+Compass	Laser+Compass	ToA/ToF	ToA/ToF	Geom. Ranging	Geom. Algorithm	C	I-L	E	PA
	[123]	IMU, Wi-Fi	Bluetooth	DR, RSSI	RSSI	PDR-B. M., RSSI-B. M.	P. Filter	N/S	I-L	S	PA
	[122]	Wi-Fi	Wi-Fi	ToA/ToF	ToA/ToF	Coop. Algorithm	Semidefinite Programming	C	I-L	S	PA+CC
	[121]	IMU	UWB	DR	RSSI	Coop. Algorithm	Info. Filter	N/S	I-L	E	PA
2016	[120]	IMU	UWB	DR	RSSI	PDR-B. M.	P. Filter	D	I-L	E	PA+R
	[119]	Wi-Fi	Wi-Fi	F. printing	ToA/ToF	KNN	Max. Grad. Descendent	D	I-L	S	PA
	[118]	IMU, Wi-Fi	Wi-Fi, Acoustic	DR, F. printing	RSSI	PDR-B. M., KNN	P. Filter	C	W/I	S+E	PA
	[117]	IMU	RFID	DR	RSSI	PDR-B. M.	P. Filter	C	W/I	E	PA
	[116]	Wi-Fi	Other RF	F. printing	RSSI	Kullbakc-Leibler Div.	Multidimensional Scaling	C	W/I	E	PA+R
	[115]	UWB	UWB	TDoA	RSSI	Coop. Algorithm	EKF	C	I-L	S	PA+PP
2015	[150]	UWB	UWB	RSSI	Multiphath C.	Ranging	EKF	N/S	N/S	S	PA+CC+R
	[114]	Wi-Fi	Wi-Fi	F. printing	RSSI	KNN	Self-organized map	D	I-L	S	PA
	[42]	Wi-Fi	Wi-Fi, Bluetooth	RSSI	RSSI	Ranging	Trilateration	C	I-L	E	PA+E
	[113]	LTE	LTE	UTDoA	UTDoA	Ranging	NLLS	C	I-L	S	PA
	[105]	Wi-Fi	Wi-Fi	ToA/ToF	ToA/ToF	Coop. Algorithm	Semidefinite Programming	N/S	N/S	S	PA
	[111]	UWB	UWB	RSSI	RSSI	Ranging	Simulated Annealing	N/S	W/I	S	PA
	[110]	IMU	Wi-Fi	DR	RSSI	PDR-B. M.	Semidefinite Programming	N/S	I-L	S	PA
	[109]	Wi-Fi	Wi-Fi	ToA/ToF	ToA/ToF	Ranging	B. Propagation	D	W/I	E	PA+CC
	[108]	Wi-Fi	Bluetooth	F. printing	RSSI	KNN	Edge Spring M.	C	I-L	S	PA
	[41]	Wi-Fi	Bluetooth	RSSI	RSSI	RSSI-B. M.	Trilateration	C	I-L	E	PA
2014	[107]	IMU	Acoustic	DR	Pos. Sharing	PDR-B. M.	EKF	D	I-L	E	PA
	[106]	IMU	UWB	DR	RSSI	PDR-B. M.	EKF	N/S	I-L	S	PA
	[40]	Wi-Fi	Wi-Fi	RSSI	RSSI	RSSI-B. M.	Trilateration	C	I-L	E	PA
	[105]	Wi-Fi	Wi-Fi	RSSI	RSSI	Coop. Algorithm	D. Stochastic Approx.	D	W/I	S	PA
	[104]	Wi-Fi	Bluetooth	RSSI	RSSI	Multilatration	Max. Grad. Descendent	N/S	I-L	S	PA
	[45]	Wi-Fi	Bluetooth	ToA/ToF	ToA/ToF	Trilateration	LS	D	I-L	S+E	PA
	[103]	Wi-Fi	Wi-Fi	RSSI	RSSI	RSSI-B. M.	Self-organazed map	N/S	W/I	S	PA
2013	[102]	IMU, Wi-Fi	Wi-Fi	DR, F. printing	RSSI, F. printing	PDR-B. M., F. printing-B. M.	P. Filter	N/S	I-L	S	PA
	[101]	IMU	Acoustic	DR	RSSI	PDR-B. M.	KF	D	I-L	N/S	PA
	[100]	Wi-Fi	Wi-Fi	F. printing	RSSI	F. printing-B. M.	Likelihood func.	C&D	I-L	S	PA
	[99]	UWB	UWB	RSSI	RSSI	Coop. Algorithm	Non-Parametric B. Propagation	N/S	N/S	S	PA+CC
	[98]	IMU, Wi-Fi	Wi-Fi	DR, F. printing	RSSI	PDR-B. M., F. printing-B. M.	P. Filter	D	I-L	S+E	PA+R
2012	[97]	IMU	Bluetooth	DR	RSSI	PDR-B. M.	Bayessian F.	D	I-L	E	PA
	[47]	IEEE.802.15.4a.CSS	IEEE.802.15.4a.CSS	TWR	TWR	Ranging	B. Propagation	C	W/I	E	PA+R
	[96]	UWB	UWB	ToA/ToF	Multipath C.	Coop. Algorithm	B. Porpagation	C/D	I-L	S	PA
2011	[95]	Wi-Fi	Wi-Fi	F. printing	F. printing	Coop. Algorithm	Self-organized map	D	I-L	S	PA+CC
	[50]	Wi-Fi	Wi-Fi	AoA, ToA/ToF	AoA, ToA/ToF	Geom. Ranging	GWF	N/S	I-L	S	R
	[94]	IEEE.802.15.4a.CSS	IEEE.802.15.4a.CSS	RSSI	RSSI	Multilateration	Trilateration	D	W/I	E	PA
	[93]	Wi-Fi	Wi-Fi	RSSI	RSSI	Ranging	P. Filter	N/S	N/S	S	PA
	[92]	IMU	Magnetic Resonant S.	DR	RSSI	PDR-B. M.	Probabilistic D. Distrib.	D	I-L	N/S	PA
	[91]	Camera	Bluetooth	QR Code	RSSI	QR Code Recongnition	Devaluation Func.	D	W/I	E	PA
	[90]	Wi-Fi	Wi-Fi	RSSI	RSSI	Coop. Algorithm	Coalitional Game	D	N/S	S	PA+CC
2010	[89]	Wi-Fi	Wi-Fi	RSSI	RSSI	RSSI-B. M.	P. Filter	C	N/S	S	PA+CC
	[88]	Wi-Fi	Wi-Fi	RSSI	RSSI	Coop. Algorithm	NLLS	D	I-L	E	PA+CC
2009	[87]	Wi-Fi	Bluetooth	F. printing	Pos. Sharing	Max. Shared Border	Max. shared Border	D	I-L	N/S	PA+CC
2007	[86]	UWB	UWB	ToA/ToF	ToA/ToF	Multilateration	Rec. Pos. Est.	D	I-L	S	PA
	[85]	Wi-Fi	Wi-Fi	RSSI	RSSI	Coop. Algorithm	NLLS	D	I-L	E	PA
2006	[83]	Wi-Fi	Wi-Fi	F. printing	RSSI	F. printing-B. M.	P. Filter	D	I-L	E	PA
	[84]	Wi-Fi	Wi-Fi	RSSI	RSSI	RSSI-B. M.	D. Loc-coverage	C	I-L	S	PA
